# Co-fermentation of cellobiose and xylose by mixed culture of recombinant *Saccharomyces cerevisiae* and kinetic modeling

**DOI:** 10.1371/journal.pone.0199104

**Published:** 2018-06-25

**Authors:** Yingying Chen, Ying Wu, Baotong Zhu, Guanyu Zhang, Na Wei

**Affiliations:** 1 Department of Civil and Environmental Engineering and Earth Sciences, University of Notre Dame, Notre Dame, Indiana, United States of America; 2 Department of Civil and Environmental Engineering, University of Pittsburgh, Pittsburgh, Pennsylvania, United States of America; Hubei University, CHINA

## Abstract

Efficient conversion of cellulosic sugars in cellulosic hydrolysates is important for economically viable production of biofuels from lignocellulosic biomass, but the goal remains a critical challenge. The present study reports a new approach for simultaneous fermentation of cellobiose and xylose by using the co-culture consisting of recombinant *Saccharomyces cerevisi*ae specialist strains. The co-culture system can provide competitive advantage of modularity compared to the single culture system and can be tuned to deal with fluctuations in feedstock composition to achieve robust and cost-effective biofuel production. This study characterized fermentation kinetics of the recombinant cellobiose-consuming *S*. *cerevisiae* strain EJ2, xylose-consuming *S*. *cerevisiae* strain SR8, and their co-culture. The motivation for kinetic modeling was to provide guidance and prediction of using the co-culture system for simultaneous fermentation of mixed sugars with adjustable biomass of each specialist strain under different substrate concentrations. The kinetic model for the co-culture system was developed based on the pure culture models and incorporated the effects of product inhibition, initial substrate concentration and inoculum size. The model simulations were validated by results from independent fermentation experiments under different substrate conditions, and good agreement was found between model predictions and experimental data from batch fermentation of cellobiose, xylose and their mixtures. Additionally, with the guidance of model prediction, simultaneous co-fermentation of 60 g/L cellobiose and 20 g/L xylose was achieved with the initial cell densities of 0.45 g dry cell weight /L for EJ2 and 0.9 g dry cell weight /L SR8. The results demonstrated that the kinetic modeling could be used to guide the design and optimization of yeast co-culture conditions for achieving simultaneous fermentation of cellobiose and xylose with improved ethanol productivity, which is critically important for robust and efficient renewable biofuel production from lignocellulosic biomass.

## Introduction

Lignocellulosic biomass has the potential to contribute substantially to future global energy demands because it is low in cost, is available at large-scale, does not compete with food production, and has high potential to reduce greenhouse gas emission [1–4]. Biorefineries, where lignocellulosic biomass materials (e.g. agricultural and forest residues, industrial wastes or energy crops) are converted to fuels and commodity products, are of increasing importance as an alternative to conventional oil refineries [5,6]. Lignocellulosic biomass, primarily composed of cellulose, hemicellulose and lignin, can be hydrolyzed chemically or enzymatically to generate pentoses and hexoses, which can be used as substrates to produce biofuel by microbial fermentation [7,8]. However, incomplete and inefficient conversion of mixed sugars in cellulosic hydrolysates into biofuels has hindered cost-effective processes [9–11].

The yeast *Saccharomyces cerevisiae*, a widely used platform microorganism for the development of renewable biofuels, has numerous advantages such as high sugar consumption rates, osmotolerance, robustness to industrial environmental conditions, and genetic tractability [12–16]. However, *S*. *cerevisiae* cannot natively ferment the hemicellulose portion of the cellulosic feedstock, for example xylose [17,18]. Extensive research efforts have been made to engineer *S*. *cerevisiae* to use xylose [9,13,19–25], in most cases by introducing either the oxidoreductase pathway (i.e. xylose reductase (XR) and xylitol dehydrogenase (XDH) or the isomerization pathway (i.e. xylose isomerase (XI) [22,26–28]. However, xylose metabolism is repressed in the presence of glucose, and the “glucose repression” makes it difficult to realize efficient fermentation using xylose and glucose mixture [19,29]. Previous studies demonstrated that heterologous expression of an intracellular cellobiose hydrolysis pathway consisting of a cellodextrin transporter gene (*cdt-1*) and an intracellular β-glucosidase gene (*gh1-1*) from *Neurospora crassa* allowed cellobiose assimilation in *S*. *cerevisiae* [30–32]. This strategy hydrolyzed cellobiose inside the yeast cell, thus alleviating glucose repression problem and enabling co-fermentation of xylose and cellobiose by engineered *S*. *cerevisiae* [30,33]. Simultaneous co-fermentation of cellobiose and xylose could increase ethanol productivity compared to the two-stage fermentation of glucose and xylose [30].

**Fig 1:**
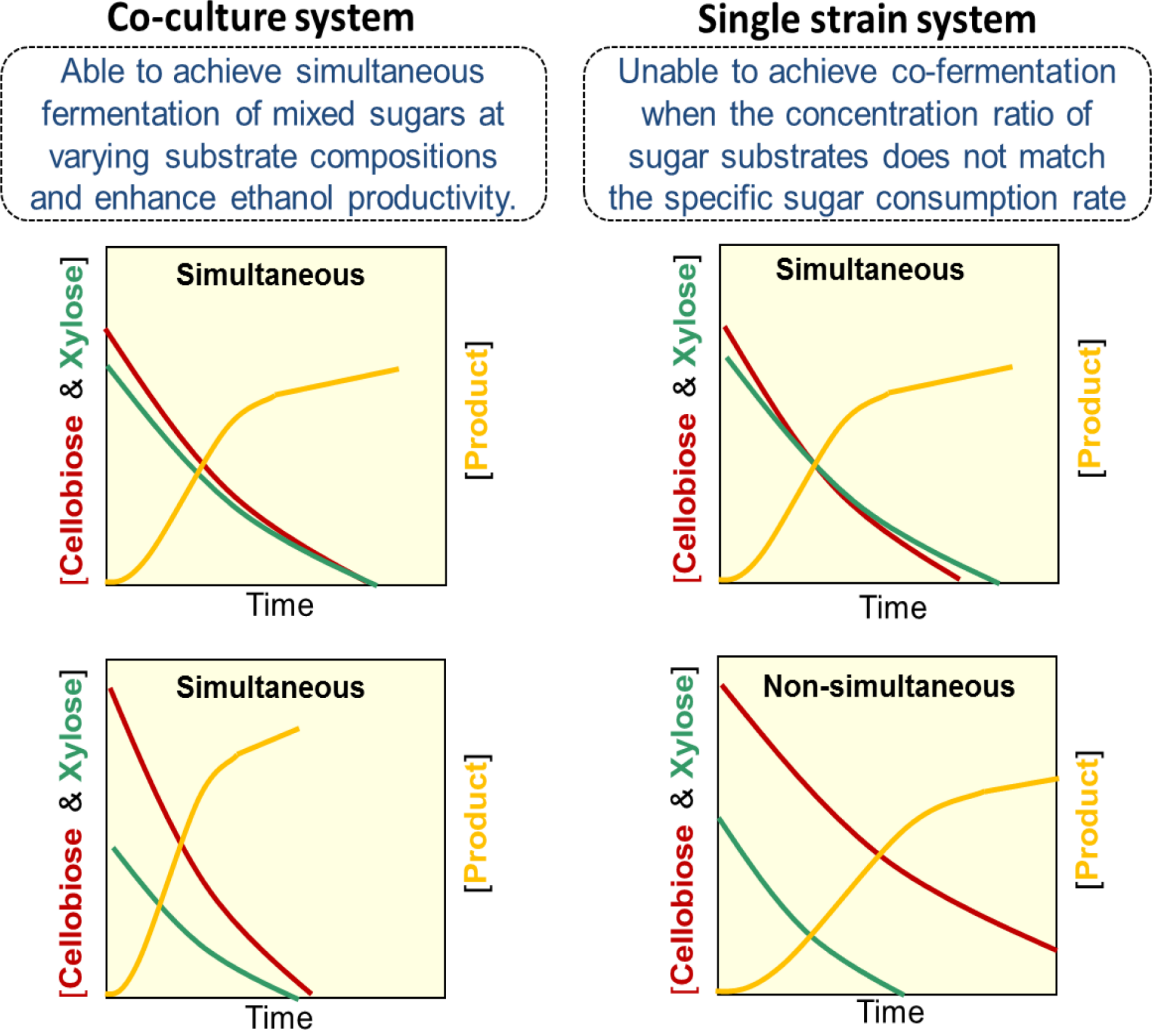
Co-culture of two specialist strains. Co-culture is adjustable to achieve simultaneous fermentation of cellobiose and xylose at different substrate concentration ratios, while single recombinant strain is unable to deal with varying substrate compositions.

While most previous metabolic engineering efforts have been focused on expanding the substrate range of *S*. *cerevisiae* to cellulosic sugars and integrating multiple substrate utilizing pathways into a single strain [9], an alternative emerging approach is engineering co-culture consortia for mixed sugar fermentation [34]. It has been observed that introducing multiple heterologous pathways into a single fermentative microorganism could lead to detrimental metabolic burden especially under high sugar concentrations [34,35]. Compared with the commonly used one-strain system, the co-culture system distributes the metabolic burden on each specialist strain and reduces the metabolic stress [36]. Furthermore, the co-culture system can provide competitive advantage of modularity compared to the single culture system. Particularly, lignocellulosic feedstocks from agricultural and industrial wastes or dedicated energy crops have diverse compositions of carbohydrates [4,9], and varied pretreatment and enzymatic hydrolysis processes would yield hydrolysates with different concentrations of sugar components [4,37]. Therefore, it is critically important that the microbial fermentation process is able to deal with fluctuations in feedstock compositions to achieve efficient and cost-effective biofuel production. As illustrated in Fig 1, the co-culture system consisting of specialist strains could be easily tuned to respond to varying concentration ratios of sugar components in the fermentation feedstock and realize simultaneous fermentation of mixed sugars, which is essential for achieving high ethanol productivity (i.e. ethanol production rate).

Considering the potential benefits of using a co-culture system, we proposed using the co-culture consisting of recombinant *S*. *cerevisiae* strains specialized in cellobiose fermentation or xylose fermentation respectively for achieving simultaneous fermentation of cellobiose and xylose mixture. While previous study has demonstrated co-fermentation of cellobiose and xylose by a single recombinant strain [30] under certain initial sugar concentration conditions, simultaneous fermentation of the two sugars would not be achieved if the concentration ratio of the two sugars does not match with the specific substrate consumption rates (which are inherent and cannot be changed when using a specific recombinant strain). The proposed co-culture approach is innovative and holds promise to address the above challenge as the composition of the co-culture (*i*.*e*. biomass of each specialist strain) is tunable; additionally, the modularity of the co-culture system would allow optimization of the process to increase ethanol productivity. However, co-fermentation of cellobiose and xylose by a co-culture system remains underexplored. Compared with pure culture, co-culture systems are complex considering the potential interactions between microbial members. It remains a critical challenge to design robust co-culture systems for efficient fermentation of mixed cellulosic sugars to produce renewable biofuels and bioproducts.

To achieve simultaneous utilization of different sugar substrates in a co-culture system, it is critically important to understand the growth and fermentation kinetics of each specialist strain when they are incubated individually or together. Kinetic models can be used to capture the characteristics of cell growth, substrate utilization and biofuel production [38,39]. The present study aimed to develop kinetic models that characterize the batch fermentation performance of the previously reported recombinant cellobiose-consuming *S*. *cerevisiae* strain EJ2 [40], xylose-consuming *S*. *cerevisiae* strain SR8 [28,33] and their co-culture. The model simulations were validated by results from independent fermentation experiments under different substrate conditions. The validated models can provide basis for optimizing the co-culture system for efficient co-fermentation of cellobiose and xylose with high ethanol productivity.

## Materials and methods

### Strains, media, and culture conditions

The recombinant *S*. *cerevisiae* strain EJ2, capable of cellobiose fermentation, was constructed through heterologous expression of β-glucosidase (encoded by *gh1-1*) and cellodextrin transporter (encoded by *cdt-1*) from *N*. *crassa* in *S*. *cerevisiae* D452-2 followed by evolutionary engineering [40]. The recombinant *S*. *cerevisiae* strain SR8, capable of fermenting xylose efficiently, was constructed through heterologous expression of *XYL1* (coding for XR), *XYL2* (coding for XDH) and *XYL3* (coding for XK) from *Scheffersomyces stipitis* in *S*. *cerevisiae* D452-2 (*MATα leu2 ura3 his3 and can1*), and optimization of expression levels of XR, XDH and XK, laboratory evolution on xylose and deletion of *ALD6* coding for aldehyde dehydrogenase [28]. The *S*. *cerevisiae* strain ES, capable of fermenting cellobiose and xylose, was created by mating EJ2 and SR8. These yeast strains were kindly provided by Dr Yong-Su Jin’s lab. The yeast strains were routinely cultivated at 30°C in Yeast Peptone (YP) medium (10 g/L of yeast extract, 20 g/L of peptone) with 20 g/L of glucose.

### Fermentation experiments

Yeast cells were grown in YP medium containing 20 g/L of glucose at 30°C to prepare inoculum for fermentation experiments. The cells were harvested at stationary phase and inoculated after being washed twice with sterilized water. Fermentation experiments under oxygen-limited conditions were performed using 20 mL of YP medium containing cellobiose and/or xylose (ranging from 10 g/L to 120 g/L) in a 125 mL Erlenmeyer flask at 30°C and 100 rpm. Culture samples were taken from fermentation experiments to measure the cell growth and metabolite concentrations. Yeast cell dry weight was determined using a microwave oven based method as described previously [37]. All fermentations were performed in duplicate.

### Analytical methods

Cell growth was monitored by OD_600_ using a UV-visible spectrophotometer (Thermo Fisher Scientific Inc., Waltham, MA). The cellobiose, xylose, and ethanol concentrations were determined by a high performance liquid chromatography (Agilent Technologies 1200 Series) equipped with a refractive index detector. A Rezex ROA-Organic Acid H+ (8%) column (Phenomenex Inc., Torrance, CA) was used. The column was eluted with 0.005N H_2_SO_4_ as a mobile phase at a flow rate of 0.6 mL/min at 50°C.

## Nomenclature

μ-Specific growth rate (/h); μ_m_-Maximum specific growth rate (/h); K_s_-Monod constant, for growth on cellobiose or xylose (g/L); K_i_-Inhibition constant, for growth on cellobiose or xylose (g/L); P-Ethanol concentration (g/L); P_m_-Ethanol concentration above which cells do not grow (g/L); β-A constant indicating the relationship between μ and P; Y_P/S_-Product yield constant; Y_X/S_-Cell yield constant from substrate; m-Maintenance coefficient (/h); v-Specific rate of ethanol formation (/h); v_m_-Maximum specific rate of product formation (/h); K_s_’-Monod constant, for product formation on cellobiose or xylose (g/L); K_i_’-Inhibition constant, for product formation on cellobiose or xylose (g/L); P_m_’-Ethanol concentration above which cells do not produce ethanol (g/L); **γ**-A constant indicating the relationship between v and P; X- Cell concentration in the mixed culture (g/L).

### Model development

To construct a mathematical model that describes the fermentation kinetics, a comprehensive kinetic model modified from the Monod kinetics was proposed. The microbial growth on each sugar is represented by the specific growth rate of the recombinant *S*. *cerevisiae* with cellobiose or xylose as the single carbon source respectively. Meanwhile, as cell metabolism was affected by substrate and product inhibition, the modified model therefore included substrate and ethanol inhibition terms. The substrate consumption equation considers substrate used for the biomass production, the ethanol production and the cell maintenance. These relationships were described by the Eqs (1)–(3) below. The basis for these equations was taken from a previous model developed for glucose and xylose fermentation by recombinant *S*. *cerevisiae* [41].

$$Cell\;growth\;\mu =\frac{1}{X}\frac{dX}{dt}=\frac{\mu_{m}S}{K_{s}+S+S^{2}/K_{i}}\left\{1-{\left(\frac{P}{P_{m}}\right)}^{\beta }\right\}$$ (1)

$$Substrate\;consumption\;-\frac{dS}{dt}=\frac{1}{Y_{P/S}}\frac{dP}{dt}+\frac{1}{Y_{X/S}}\frac{dX}{dt}+mX$$ (2)

$$Ethanol\;production\;v=\frac{1}{X}\frac{dP}{dt}=\frac{v_{m}S}{{K_{s}}^{\prime }+S+S^{2}/{K_{i}}^{\prime }}\left\{1-{\left(\frac{P}{{P_{m}}^{\prime }}\right)}^{r}\right\}$$ (3)

The terms used are defined fully in the Nomenclature section. Initial estimations of some parameter values were based on experimental data and literature reports, which provided a basis for determining the final parameter values. Specifically, the parameters, μ_m_ and K_s_, were tentatively estimated from the experimental data during the log growth phase by using Lineweaver-Burk plot based on Monod equation. The value for P_m_ was obtained experimentally. The initial estimation of Y_P/S_ was based on the literature [28,40]. The initial values of other parameters were tentatively estimated by manual adjustment for a good visual fit with the experimental data. The initial guesstimates of kinetic parameters were then used as the input to run the program iterations in MATLAB 9.1.0 (The MathWorks, Inc.) to obtain the final best-fit values of the parameters using the least-square method by minimization of the total Residue Sum of Square (RSS) and Root Mean Square Error (RMSE) of experimental and fitted values.

## Results

### Simulation of cellobiose fermentation by *S*. *cerevisiae* EJ2

**Fig 2:**
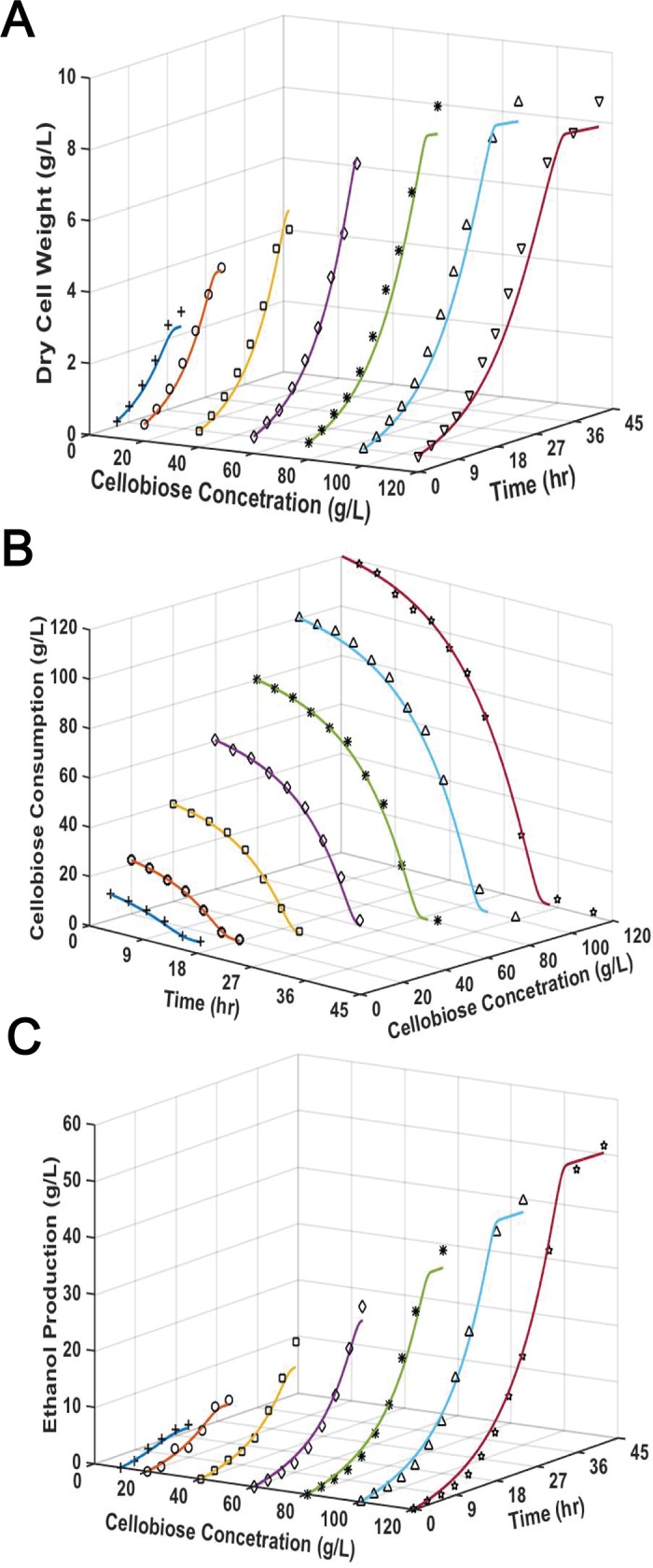
Cellobiose fermentation by *S*. *cerevisiae* EJ2. Experimental and model simulated profiles of (A) cell growth, (B) cellobiose consumption and (C) ethanol production at different initial cellobiose concentrations for *S*. *cerevisiae* strain EJ2. Lines represent model predictions and symbols represent the means of duplicate experimental results.

Batch experiments were conducted to characterize the cellobiose fermentation performance of *S*. *cerevisiae* EJ2 with various initial sugar concentrations ranging from 10 g/L to 120 g/L (Fig 2). The profiles of cell growth, cellobiose consumption and ethanol production were simulated to establish the cellobiose fermentation model. The model was a non-linear model with multi-parameters, and initial estimation of parameters values is needed to obtain the optimized parameter values. The initial values for some of the parameters could be obtained from experimental data and/or literature reports. Herein, the initial values for μ_max_ and K_S_ were estimated based on the cell growth data from the fermentation experiments. The specific cell growth rates of EJ2 increased from 0.146 h^-1^ to 0.152 h^-1^ when cellobiose concentrations increased from 10 g/L to 40 g/L. The specific growth rate started to decrease when initial cellobiose concentration was higher than 60 g/L, which might be due to substrate inhibition and osmotic stress [42]. The μ values under conditions with cellobiose lower than 60 g/L were fitted in Monod model by using Lineweaver-Burk plot to determine μ_max_ and K_S_. It was found that μ_max_ and K_S_ were 0.154 h^-1^ and 0.568 g/L, respectively. The Y_P/S_ of 0.5 g/g for EJ2 was obtained from previous report [40]. The P_m_, describing the ethanol concentration that completely inhibits the cell growth in the Luong model could be obtained experimentally. To determine the tentative P_m_ value, cell growth experiments with a range of initial ethanol concentrations (0–120 g/L) were performed. The initial cellobiose concentration of 40 g/L was used in the experiments as no substrate inhibition was observed under this substrate condition. The effect of ethanol concentration on the growth of EJ2 was shown in S1A Fig, and the results indicated that the value for P_m_ is in the range of 60 g/L- 90 g/L. Further experiment showed that there was a complete inhibition of cell growth when ethanol concentration was greater than 70 g/L.

Other parameters besides μ_max_, K_S_, Y_P/S_ and P_m,_ in the models were estimated based on the least square methods and optimized by Newton method, by using ODE45 function in MATLAB,. In the model development, the initial sugar concentration was found to have a substantial impact on ethanol productivity. Thus, a term that takes into account of the initial sugar concentration was incorporated into the Eq (3), yielding an empirical correlation Eq (4) for simulating ethanol production.

$$v=exp(0.011\times S_{0})\frac{v_{m}S}{{K_{s}}^{\prime }+S+S^{2}/{K_{i}}^{\prime }}\left\{1-{\left(\frac{P}{{P_{m}}^{\prime }}\right)}^{r}\right\}$$ (4)

The model parameters were summarized in Table 1. As shown in Fig 2, the model demonstrates excellent simulation of the experimental data for cell growth, cellobiose consumption and ethanol production (RMSE<10) in cellobiose fermentation by EJ2.

**Table 1:** Kinetic model parameters of cellobiose and xylose fermentation by *S*. *cerevisiae* EJ2 and SR8, respectively.

Modules	Parameter	Cellobiose (EJ2)	Xylose (SR8)
Cell growth	μ_m_ (/h)	0.154	0.154
	K_s_ (g/L)	0.568	1.31
	K_i_ (g/L)	204	N.A.
	P_m_ (g/L)	69	25.33
	β	1.1	0.742
	m (/h)	0.01	0.01
Substrate consumption	Y_P/S_ (g/g)	0.5	0.4
	Y_X/S_ (g/g)	0.48	0.35
	b1	N.A.	1.12
	b2	N.A.	1.32
Ethanol production	v_m_ (/h)	0.416	0.401
	K_s_’ (g/L)	5	13.32
	K_i_’ (g/L)	52	N.A.
	P_m_’ (g/L)	100	27
	**γ**	1.1	1.04

N.A.: Not Applicable

### Simulation of xylose fermentation by *S*. *cerevisiae* SR8

**Fig 3:**
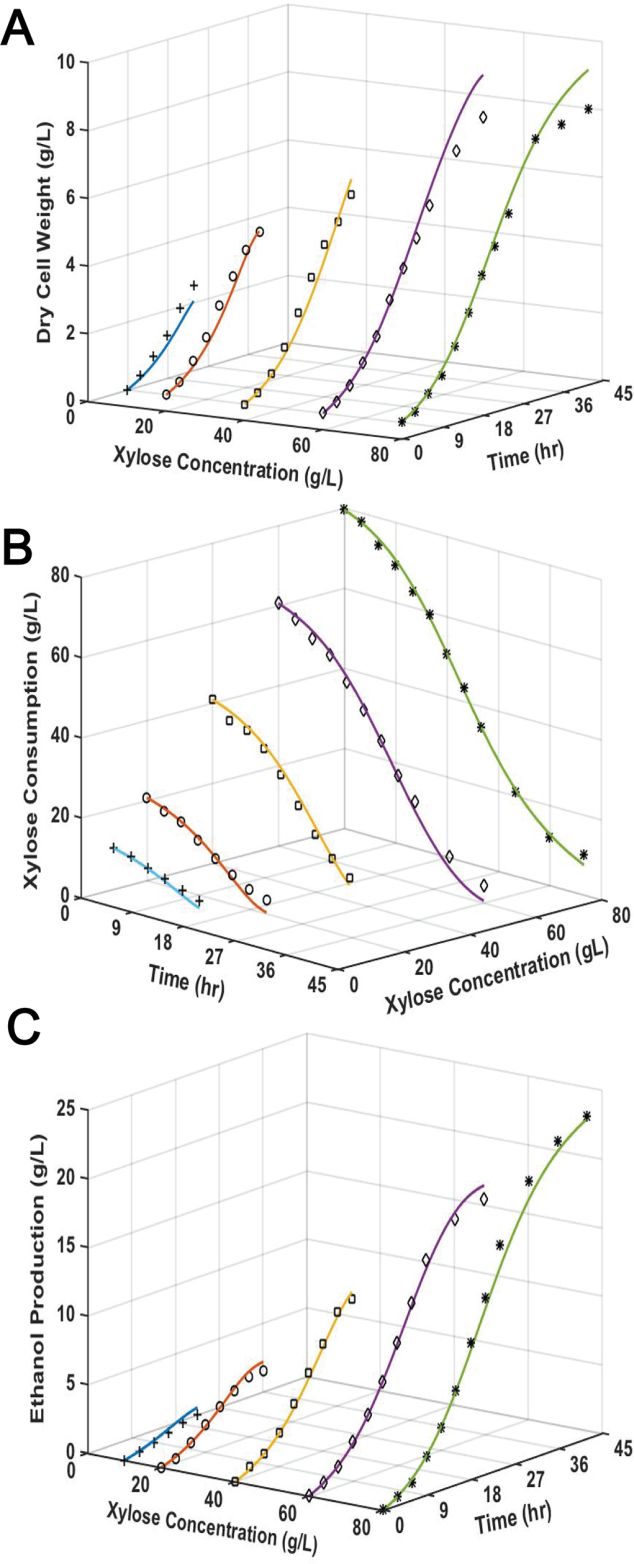
Xylose fermentation by *S*. *cerevisiae* SR8. Experimental and model simulated profiles of (A) cell growth, (B) xylose consumption and (C) ethanol production at different initial xylose concentrations using *S*. *cerevisiae* strain SR8. Lines represent model predictions and symbols represent the means of duplicate experimental results.

Batch experiments were conducted to characterize the xylose fermentation performance of SR8 with various initial sugar concentrations ranging from 10 g/L to 80 g/L (Fig 3). The profiles of cell growth, xylose consumption and ethanol production were simulated to establish the xylose fermentation model. Following the same protocol of simulating cellobiose fermentation by EJ2, the determination of μ_max_, K_S_, Y_P/S_ and P_m_ were preceded by the estimation of other kinetic parameters. The specific cell growth rates of SR8 increased from 0.136 h^-1^ to 0.149 h^-1^ when xylose concentrations increased from 10 g/L to 40 g/L. Based on the experimental data regressed by Lineweaver-Burk plot, the maximum growth rate μ_max_ was 0.154 h^-1^ and K_S_ was 1.31 g/L, respectively. The Y_P/S_ of 0.35 g/g for SR8 was obtained from previous report [28]. Afterwards, the effect of ethanol on the cell growth was studied to determine the P_m_ value for xylose fermentation. To determine the tentative P_m_ value, cell growth experiments with a range of initial ethanol concentrations (0–120 g/L) were performed. The initial xylose concentration of 40 g/L was used in the experiments as no substrate inhibition was observed under this condition. The effect of ethanol on the growth of SR8 was shown in S1B Fig, and the results indicated that the value for P_m_ is in the range of 60 g/L- 90 g/L. Further experiment showed that there was a complete inhibition of cell growth when ethanol concentration was greater than 85 g/L.

Noticeably, SR8 was unable to completely uptake xylose under all substrate conditions when xylose concentration decreased to a certain level (data not shown). This could be attributed to non-fermentative feature of xylose [43]. Assimilation of ethanol became dominant after xylose consumption stopped, which could result in discrepancy of ethanol production between model prediction and experimental data [44]. Therefore the model simulation did not account for the experimental data when xylose concentration leveled off. Additionally, to tentatively determine the maximum ethanol production P_m_’, ethanol concentration above which cells do not produce ethanol, the ethanol production under a broad range of xylose concentrations (10 g/L-120 g/L) was studied. The results indicated that increasing initial concentration of xylose from 100 g/L to 120 g/L did not lead to increase of ethanol production at the end of fermentation and the parameter P_m_’ was estimated to be 27 g/L. Interestingly, the experimental results showed P_m_’ value was much lower than P_m_, which might be due to the consumption of ethanol by SR8 and more is discussed in Discussion section.

Other parameters besides μ_max_, K_S_, Y_P/S,_ P_m_ and P_m_’ in the models were estimated based on the least square methods and optimized by Newton method, by using ODE45 function in MATLAB. In our initial model development, the experimentally estimated P_m_ and P_m_’ were directly plugged into the model equation, but we failed to simulate the biomass accumulation and ethanol concentration profiles. Therefore, we incorporated the effect of ethanol consumption by SR8 by using the P_m_ value obtained by curve fitting and by introducing two constants b1 and b2 to amend the Y_X/S_ and Y_P/S_, respectively. Additionally, since the substrate inhibition effect was not as significant as ethanol inhibition and the ignorance of substrate inhibition terms would actually lead to the minimization of total RMSE (S1 Table), we ignored the substrate inhibition constant K_i_ and K_i_^’^. The xylose kinetic model is modified and shown in Eqs (5)–(7).

$$Cell\;growth\;\mu =\frac{1}{X}\frac{dX}{dt}=\frac{\mu_{m}S}{K_{s}+S}\left\{1-{\left(\frac{P}{P_{m}}\right)}^{\beta }\right\}$$ (5)

$$Substrate\;consumption\;-\frac{dS}{dt}=\frac{1}{b1\times Y_{P/S}}\frac{dP}{dt}+\frac{1}{{b2\times Y}_{X/S}}\frac{dX}{dt}+mX$$ (6)

$$Ethanol\;production\;v=\frac{1}{X}\frac{dP}{dt}=\frac{v_{m}S}{{K_{s}}^{\prime }+S}\left\{1-{\left(\frac{P}{{P_{m}}^{\prime }}\right)}^{r}\right\}$$ (7)

The model parameters were summarized in Table 1. As shown in Fig 3, the model demonstrates good simulation of the experimental data for cell growth, xylose consumption and ethanol production (RMSE<15).

### Simultaneous fermentation of cellobiose and xylose by co-culture system

**Fig 4:**
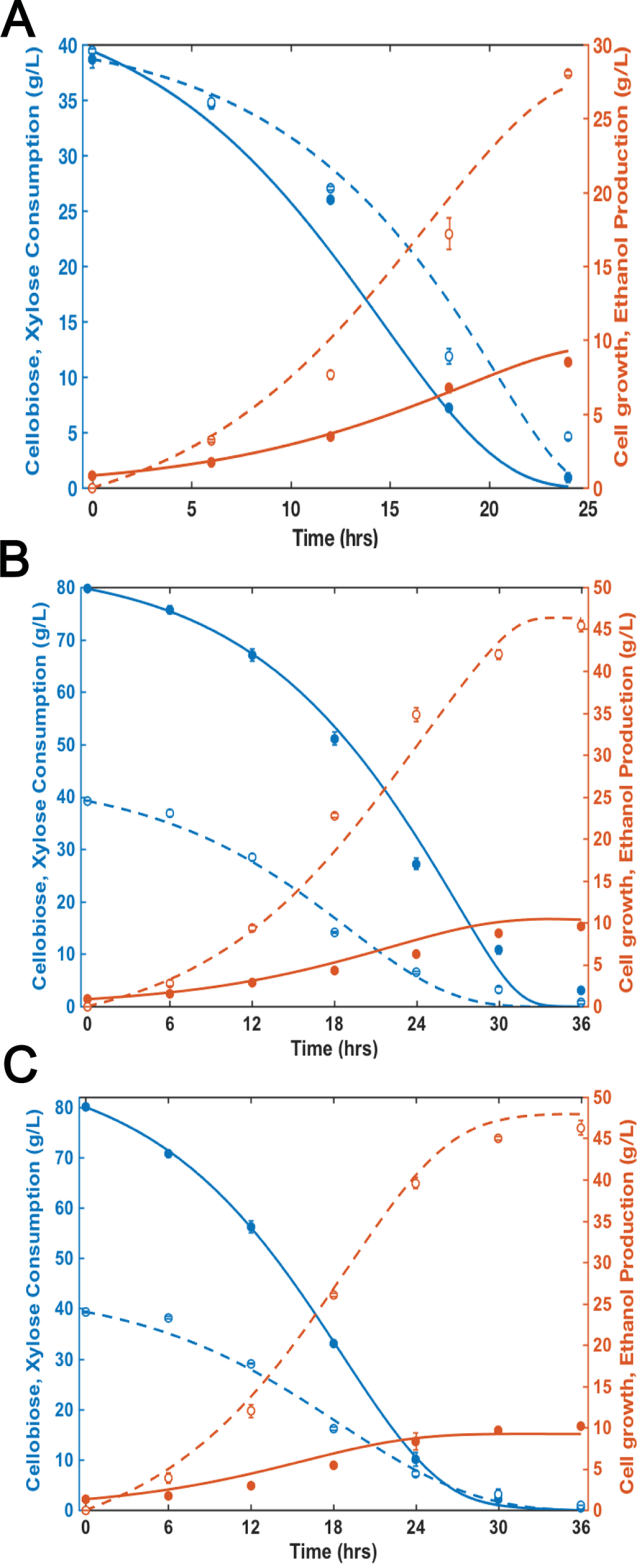
Co-fermentation of cellobiose and xylose by the co-culture system at different initial sugar concentrations and inoculum sizes using *S*. *cerevisiae* strain EJ2 and SR8. The initial sugar concentrations are 40 g/L cellobiose+40 g/L xylose (A) and 80 g/L cellobiose+40 g/L xylose (B and C). The initial cell densities of EJ2+SR8 were 0.45 g dry cell weight/L+0.45 g dry cell weight/L (A and B) and 0.45 g dry cell weight /L+0.9 g dry cell weight /L (C). Lines represent model predictions and symbols represent experimental data (blue solid line, model curve of cellobiose consumption; blue dash line, model curve of xylose consumption; red solid line, model curve of cell growth; red dash line, model curve of ethanol production; blue solid circle, cellobiose concentration (g/L); blue hallow circle, xylose concentration (g/L); red solid circle, cell density (g dry cell weight /L); red hallow circle, ethanol concentration (g/L)).Experimental results are the means of duplicate experiments; error bars indicating standard deviations are not visible when smaller than the symbol size.

Batch experiments for co-fermentation of cellobiose and xylose were conducted by using co-culture of *S*. *cerevisiae* strains EJ2 and SR8. For comparison, we also conducted co-fermentation of cellobiose and xylose by using a single-strain system with the strain ES, which was developed from mating of EJ2 and SR8 and was able to consume both cellobiose and xylose. In the fermentation of cellobiose (40 g/L) and xylose (40 g/L) with co-culture of EJ2 and SR8 at the same initial cell density, cellobiose and xylose were consumed simultaneously and fermentation of the two sugars was almost completed at the same time (Fig 4A). When the initial sugar composition changed to 80 g/L of cellobiose and 40 g/L of xylose, substantial amount of cellobiose remained unconsumed when xylose was mostly consumed after 24 hours (Fig 4B). By adjusting the initial biomass ratio of EJ2 and SR8 to 2:1 (i.e. doubling the initial biomass of EJ2), 80 g/L of cellobiose and 40 g/L of xylose were depleted at the same time (Fig 4C). The modularity of the co-culture system allowed for simultaneous depletion of the two sugars and significantly enhanced the overall ethanol productivity and sugar consumption rates (which are important for efficient and cost-effective industrial fermentation). As for the fermentation by the mated strain ES, co-fermentation of cellobiose and xylose was observed when the initial sugar concentrations were equal (i.e. 40 g/L of cellobiose and 40 g/L of xylose). However, when the concentration ratio of the two sugars changed to be 80 g/L of cellobiose and 40 g/L of xylose, the single strain system could not be manipulated to achieve simultaneous completion of cellobiose and xylose fermentation (S2 Fig). The results suggested that the co-culture system could offer the advantage of modularity to achieve simultaneous fermentation of both sugars at different concentration ratios by manipulating the biomass ratio of the two specialist strains.

We then developed the kinetic model to simulate co-fermentation of cellobiose and xylose by the co-culture system. The co-fermentation model was developed by combining the two individual yeast fermentation models (*i*.*e*. EJ2 and SR8 models). Since EJ2 and SR8 solely consumes cellobiose and xylose respectively, it is reasonable to assume that there was no competition for the sugar substrate between the two strains. It should be noted that the individual profiles of biomass and ethanol production by EJ2 and SR8 during the co-culture fermentation were unknown from the experimental data. Therefore, the terms considering the ratios of initial sugar concentrations and inoculum sizes were incorporated to both cell growth (x) and ethanol production (P). The equations were shown in equations 1–10 (see S1 Text). r1-r6 represent the weighing factors, S_o, cellobiose_ and S_o, xylose_ are the initial concentrations of cellobiose and xylose, and X_o, cellobiose_ and X_o, xylose_ are the initial inoculum sizes of EJ2 an SR8, respectively. The other terms are defined fully in the Nomenclature section.

**Fig 5:**
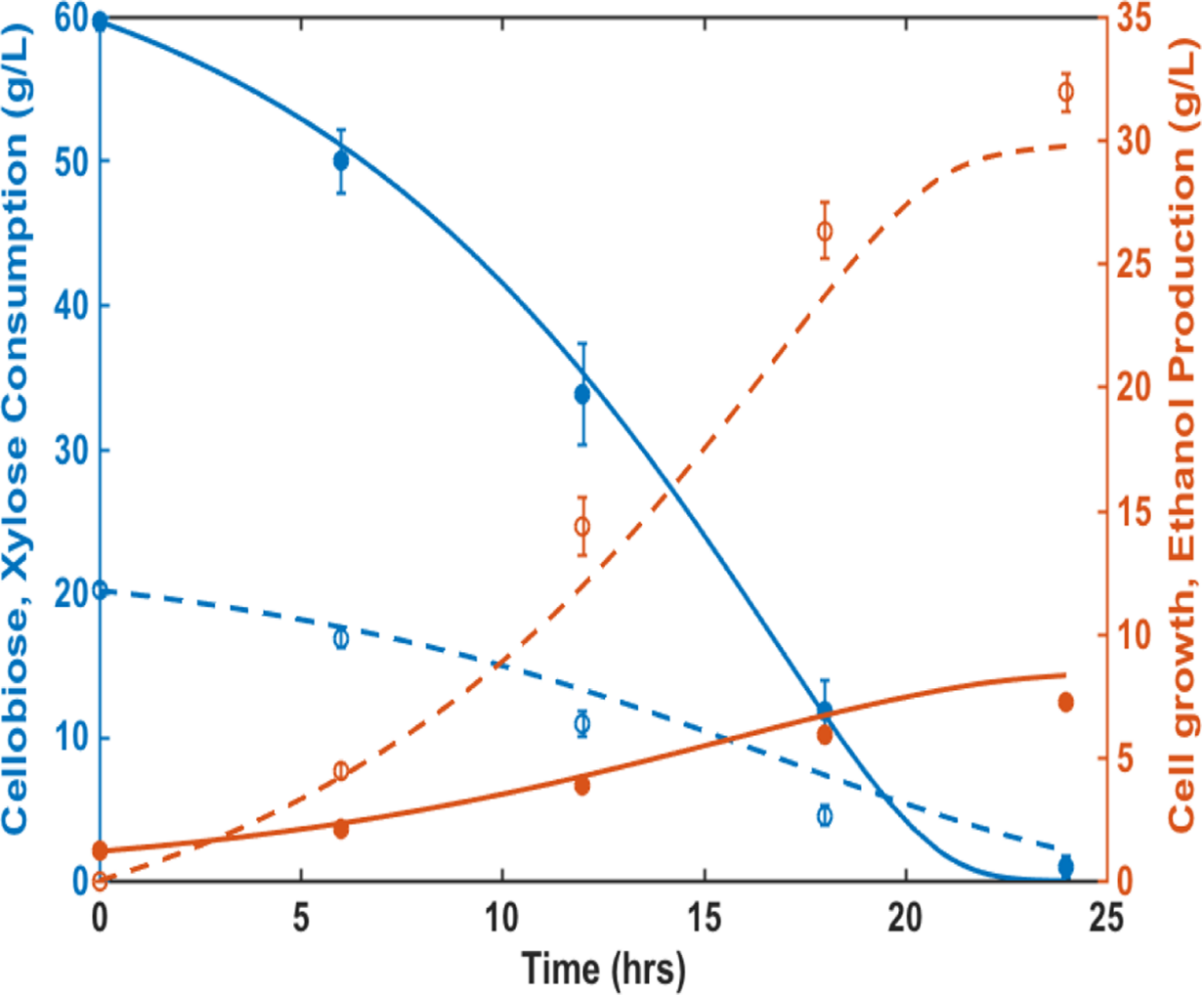
Validation of co-culture model prediction for simultaneous fermentation of cellobiose and xylose by *S*. *cerevisiae* strains EJ2 and SR8. The initial sugar concentrations are 60 g/L cellobiose+20 g/L xylose. The initial cell densities of EJ2+SR8 were 0.45 g dry cell weight /L+0.9 g dry cell weight /L. (blue solid line, model curve of cellobiose consumption; blue dash line, model curve of xylose consumption; red solid line, model curve of cell growth; red dash line, model curve of ethanol production; blue solid circle, cellobiose concentration (g/L); blue hallow circle, xylose concentration (g/L); red solid circle, cell density (g dry cell weight /L); red hallow circle, ethanol concentration (g/L)).Experimental results are the means of duplicate experiments; error bars indicating standard deviations are not visible when smaller than the symbol size.

The weighing factors were estimated based on the least square methods and optimized by Newton method, by using ODE45 function in MATLAB. Table 2 summarized the estimated values of weighing factors. The model simulations of fermentation profiles for three batch experiments with different initial sugar concentrations and inoculation sizes were shown in Fig 4. The results showed good agreement between the co-culture model prediction and the experimental data. Then we further validated the ability of the model to predict sugar consumption and ethanol production in fermentation of 60 g/L of cellobiose and 20 g/L of xylose. Fermentation profiles were simulated for conditions with three different initial cell biomass ratios of EJ2 and SR8, including 1:1, 2:1 and 3:1 (S3 Fig). The results suggested that simultaneous consumption of cellobiose and xylose could be achieved with the initial cell biomass ratio of 2:1 for EJ2 and SR8. Under this initial biomass condition, both sugars could be almost depleted in 20 hours and the overall ethanol production rate would be the highest among the three conditions. Therefore, we performed fermentation experiment with EJ2 at an initial cell density of 0.9 g dry cell weight /L and SR8 at an initial cell density of 0.45 g dry cell weight /L. The experimental data and the model simulation matched well (Fig 5). These results suggested that the model could provide guidance on how to achieve simultaneous consumption of the mixed sugars with adjustable biomass of each specialist strain in response to changing substrate concentrations.

**Table 2:** Values of weighing factors of in the model for cellobiose and xylose co-fermentation by the co-culture of *S*. *cerevisiae* EJ2 and SR8.

Parameter	Value
r1	3.75
r2	1.76
r3	1.44
r4	2.48
r5	1.82
r6	4.29

## Discussion

The results from the present study demonstrated that using co-culture of the two specialist recombinant yeast strains EJ2 and SR8 could achieve simultaneous fermentation of cellobiose and xylose and that the co-culture system could be manipulated for efficient ethanol production when the sugar composition changed. Prior research efforts have significantly advanced the co-fermentation of cellobiose and xylose by engineering *S*. *cerevisiae* strains that express various sugar consumption pathways [9,30,33,45]. However, the investigation of using mixed culture of recombinant yeast strains for simultaneous fermentation of cellobiose and xylose has not been reported before. The present study developed a kinetic model for the co-culture of *S*. *cerevisiae* strains EJ2 and SR8, and the model was able to predict batch fermentation performance by the co-culture system at different initial sugar concentrations and inoculum sizes. As the basis for the co-culture model, kinetic models representing cellobiose fermentation by EJ2 and xylose fermentation by SR8, respectively, have been developed by exploring data from pure culture fermentation experiments. The established models will contribute to designing mixed culture system with the recombinant yeast strains for efficient consumption of cellobiose and xylose derived from lignocellulosic biomass.

The co-culture system established here is distinct from previous work on co-culture mixed sugar fermentation. Previous work mostly focused on exploiting native capabilities of different microbial species for fermentation of cellulosic sugar mixtures [36,44,46,47]. For examples, ethanol production from glucose and xylose sugar mixture has been reported by using mixed culture of *Zymomonas mobilis* and *Candida tropicalis* [48], *Z*. *mobilis* and *Pichia stipitis* [49], *Z*. *mobilis* and *Pachysolen tannophilus* [50], *S*. *cerevisiae* and *Esherichia coli* [36], *S*. *cerevisiae* and *P*. *stipitis* [51], *S*. *cerevisiae* and *P*. *tannophilis* [52], and *S*. *cerevisiae* and *C*. *tropicalis* [53]. The co-culture systems consisting of heterogeneous microbial species required the cultivation conditions to be carefully designed so as to meet the growth and metabolic needs of each microorganism, which could be challenging and sometimes compromises the fermentation performance. In contrast, the co-culture system developed in this study used different engineered strains derived from one host microorganism (*i*.*e*. *S*. *cerevisiae*), which could enable each specialist strain to achieve maximum sugar consumption capability without constraints from cultivation conditions. Notably, while the model developed in this work was for xylose and cellobiose fermentation, the kinetic equations could be adapted to the fermentation processes of other sugar substrates. Also the kinetic model development process provides an example that could inspire the establishment of similar models for co-culture systems of specialist strains for mixed sugar fermentation.

The development of kinetic models for EJ2 and SR8 provided understanding of the fermentation metabolisms of the two engineered strains. The specific ethanol production rate of EJ2 during cellobiose fermentation was found to be related with initial cellobiose concentrations. This might be relevant to the accumulation of cellodextrins by EJ2. Transient accumulation of cellodextrins has been observed by EJ2 during cellobiose fermentation in previous study [9,30]. Accumulation of cellodextrin would affect the productivity as the transport rates of cellodextrins are different from that for cellobiose. The ethanol yield from cellobiose by EJ2 was found to be 98% of the theoretical yield, suggesting the effectiveness of EJ2 to convert cellobiose to ethanol. Based on the values of the model parameters Y_p/s_, P_m_ and P_m_’, we could estimate that the sugar concentrations causing substrate inhibition to terminate cell growth and ethanol production would be 138 g/L and 200 g/L, respectively, which are similar to those found in yeast glucose fermentation [41,54]. As for SR8, complete inhibition of ethanol on cell growth was found to be 85 g/L, and significant substrate inhibition to terminate cell growth would occur at a xylose concentration higher than 240 g/L. Noticeably, ethanol production from xylose fermentation by SR8 could not exceed 27 g/L even when substrate concentration was high (in the range of 80–100 g/L). A possible reason could be assimilation of ethanol by SR8. In the presence of dissolved oxygen in the culture medium, ethanol can be simultaneously metabolized with sugar substrate by the respiratory pathway in the yeast if the sugar metabolic capacity is lower than the oxidative capacity [55]. This phenomenon is more obvious in xylose fermentation than glucose fermentation [56] and has been recognized in the xylose model development [44]. Further work is needed to evaluate the oxidative capacity of the strain and experimentally quantify the assimilation of ethanol by SR8.

The co-culture model developed on the basis of the single culture models incorporates the terms to represent contribution of each specialist strain to the overall cell growth and ethanol production. The weighing factors indicated that the strain EJ2 was more sensitive to ethanol than SR8 (r1>r2) while the latter tended to dominate the biomass accumulation rate (r3<r4) and the ethanol production rate (r5<r6). Noticeably, the total ethanol production or biomass accumulation in the co-fermentation was not equal to the direct sum of the corresponding parameters derived from single strain fermentation. Instead, the ethanol production P in co-fermentation was slightly lower than the direct sum of ethanol production from single sugar fermentation by EJ2 and SR8 (i.e. P_cellobiose_+P_xylose_). For example, when cellobiose and xylose are both 40 g/L, with the reported model parameters, P_cellobiose_ + P_xylose_ = 1.38*P. This observation indicated that metabolisms of the two strains in the co-culture are not completely independent and there was a need of introducing weighing factors to consider interaction of the two strains in mixed culture in general. Potential mutual influences might include competition for nutrients in the medium and inhibition from the same product ethanol. The weighing factor r1 and r2 were introduced to amend the ethanol inhibition effect (the resulted P_cellobiose_ and P_xylose_) in the mixed culture for each specialist strain. Additionally, as shown in Eqs 3 and 4 (see S1 Text), additional terms considering initial sugar concentration and biomass were incorporated. Such modifications were valid as the developed model simulated the co-fermentation processes at various substrate composition conditions well (Fig 4 and Fig 5). Future work will focus on comprehensively understanding the potential interaction between the specialist strains and optimizing the co-culture fermentation process.

## Supporting information

S1 Text Equations for co-culture model. (DOCX)

S1 Table Sensitivity analysis of substrate inhibition effect parameters and product inhibition effect parameters in the model of xylose fermentation kinetics by *S*. *cerevisiae* SR8. Error estimation is represented by total Root Mean Square Error (RMSE).
(DOCX)

S1 Fig **Effects of initial ethanol concentrations on cell growth of *S*. *cerevisiae* (A) EJ2 and (B) SR8.** The inoculum size is 0.45 g dry cell weight/L and initial concentrations of cellobiose and xylose are 40 g/L.
(TIF)

S2 Fig Co-Fermentation of cellobiose and xylose by *S*. *cerevisiae* strain ES. Experimental profiles of cell growth, cellobiose consumption, xylose consumption and ethanol production at different initial sugar concentrations using *S*. *cerevisiae* strain ES. The initial sugar concentrations are 40 g/L cellobiose+40 g/L xylose (A) and 80 g/L cellobiose+ 40 g/L xylose (B). The inoculum size is 0.45 g dry cell weight/L. Results are the means of duplicate experiments; error bars indicating standard deviations are not visible when smaller than the symbol size.
(TIF)

S3 Fig Prediction of co-fermentation of cellobiose and xylose by co-culture. Model simulated profiles of cell growth, cellobiose consumption, xylose consumption and ethanol production at initial sugar concentrations and inoculum sizes using *S*. *cerevisiae* strain EJ2 and SR8. The initial sugar concentrations are 60 g/L cellobiose+20 g/L xylose. The initial cell densities of EJ2+SR8 were 0.45 g dry cell weight/L+0.45 g dry cell weight/L (A), 0.9 g dry cell weight /L+0.45 g dry cell weight /L (B) and 1.35 g dry cell weight /L+0.45 g dry cell weight /L (C). Lines represent model predictions (blue solid line, model curve of cellobiose consumption; blue dash line, model curve of xylose consumption; red solid line, model curve of cell growth; red dash line, model curve of ethanol production).
(TIF)
