# Supplementary material for: Co-fermentation of cellobiose and xylose by mixed culture of recombinant Saccharomyces cerevisiae and kinetic modeling
Source: PLoS One. 2018 Jun 25;13(6):e0199104. doi: 10.1371/journal.pone.0199104 (PMC6016917; doi:10.1371/journal.pone.0199104)
Supplement: S1 Text — (DOCX) [file pone.0199104.s001.docx]

**S1 Text. Equations for co-culture model**

$P_{cellobiose, m}=P_{cellobiose}\times r1\times\frac{S_{0, cellobiose}}{S_{0, cellobiose}+S_{0, xylose}}\times\frac{X_{0, cellobiose}}{X_{0, cellobiose}+X_{0, xylose}}$ (1) $P_{xylose, m}=P_{xylose}\times r2\times\frac{S_{0, xylose}}{S_{0, cellobiose}+S_{0, xylose}}\times\frac{X_{0, xylose}}{X_{0, cellobiose}+X_{0, xylose}}$ (2)

$\frac{dX_{cellobiose}}{dt}=\frac{0.154*S_{cellobiose}}{0.568+S_{cellobiose}+{S_{cellobiose}}^{2}/204}\left\{ 1-\left( \frac{P_{cellobiose,m}}{69} \right)^{1.1} \right\}\times X\times r3\times\frac{S_{0, cellobiose}}{S_{0, cellobiose}+S_{0, xylose}}\times\frac{X_{0, cellobiose}}{X_{0, cellobiose}+X_{0, xylose}}$ (3)

$\frac{dX_{xylose}}{dt}=\frac{0.154*S_{xylose}}{1.31+S_{xylose}}\left\{ 1-\left( \frac{P_{xylose,m}}{25.33} \right)^{0.742} \right\}\times X\times r4\times\frac{S_{0, xylose}}{S_{0, cellobiose}+S_{0, xylose}}\times\frac{X_{0, xylose}}{X_{0, cellobiose}+X_{0, xylose}}$ (4)

$\frac{dX}{dt}=\frac{dX_{cellobiose}}{dt}+\frac{dX_{xylose}}{dt}$ (5)

$-\frac{dS_{cellobiose}}{dt}=\frac{1}{0.5}\frac{dP_{cellobiose}}{dt}+\frac{1}{0.48} \frac{dX_{cellobiose}}{dt}+0.01*X$ (6)

$-\frac{dS_{xylose}}{dt}=\frac{1}{1.32*0.5}\frac{dP_{xylose}}{dt}+\frac{1}{1.12*0.4} \frac{dX_{xylose}}{dt}+0.01*X$ (7)

$\frac{dP_{cellobiose}}{dt}=\frac{0.416*S_{cellobiose}*exp(0.011*S_{0, cellobiose})}{5+S_{cellobiose}+{S_{cellobiose}}^{2}/52}\left\{ 1-\left( \frac{P_{cellobiose,m}}{100} \right)^{1.1} \right\}\times X\times r5\times\frac{S_{0, cellobiose}}{S_{0, cellobiose}+S_{0, xylose}}\times\frac{X_{0, cellobiose}}{X_{0, cellobiose}+X_{0, xylose}}$ (8)

$\frac{dP_{xylose}}{dt}=\frac{0.401*S_{xylose}}{13.32+S_{xylose}}\left\{ 1-\left( \frac{P_{xylose,m}}{27} \right)^{0.742} \right\}\times X\times r6\times\frac{S_{0, xylose}}{S_{0, cellobiose}+S_{0, xylose}}\times\frac{X_{0, xylose}}{X_{0, cellobiose}+X_{0, xylose}}$ (9)

$\frac{dP}{dt}=\frac{dP_{cellobiose}}{dt}+\frac{dP_{xylose}}{dt}$(10)

Note: The numeric values in the equations above were obtained from Table 1. P_cellobiose, m_-Empirical ethanol concentration fitted in the Luong model for cellobiose fermentation in the mixed culture (g/L); P_xylose, m_- Empirical ethanol concentration fitted in the Luong model for xylose fermentation in the mixed culture (g/L).
