# Supplementary material for: Co-fermentation of cellobiose and xylose by mixed culture of recombinant Saccharomyces cerevisiae and kinetic modeling
Source: PLoS One. 2018 Jun 25;13(6):e0199104. doi: 10.1371/journal.pone.0199104 (PMC6016917; doi:10.1371/journal.pone.0199104)
Supplement: S1 Table — Error estimation is represented by total Root Mean Square Error (RMSE). (DOCX) [file pone.0199104.s002.docx]

**S1 Table. Sensitivity analysis of substrate inhibition effect parameters and product inhibition effect parameters in the model of xylose fermentation kinetics by *S. cerevisiae* SR8.** Error estimation is represented by total Root Mean Square Error (RMSE).

| **Term** | **Parameter** | **Parametric value** | **Total RMSE^*^** |
| --- | --- | --- | --- |
| substrate inhibition effect | not consider | -- | 12.7 |
|  | K_i_ | 10 | 111.1 |
|  |  | 1000 | 109.0 |
|  |  | 10000 | 109.0 |
|  | K_i_’ | 10 | 111.2 |
|  |  | 1000 | 110.0 |
|  |  | 10000 | 109 |
| product inhibition effect | β | 0.0742 | 82.1 |
|  |  | 0.742 | 12.7 |
|  |  | 7.42 | 35.7 |
|  | **γ** | 0.104 | 35.8 |
|  |  | 1.04 | 12.7 |
|  |  | 10.4 | 15.6 |

^*^ Total RMSE: the sum of RMSE under conditions with different initial xylose concentrations.
