# Supplementary figures and images for: Co-fermentation of cellobiose and xylose by mixed culture of recombinant Saccharomyces cerevisiae and kinetic modeling
Source: PLoS One. 2018 Jun 25;13(6):e0199104. doi: 10.1371/journal.pone.0199104 (PMC6016917; doi:10.1371/journal.pone.0199104)

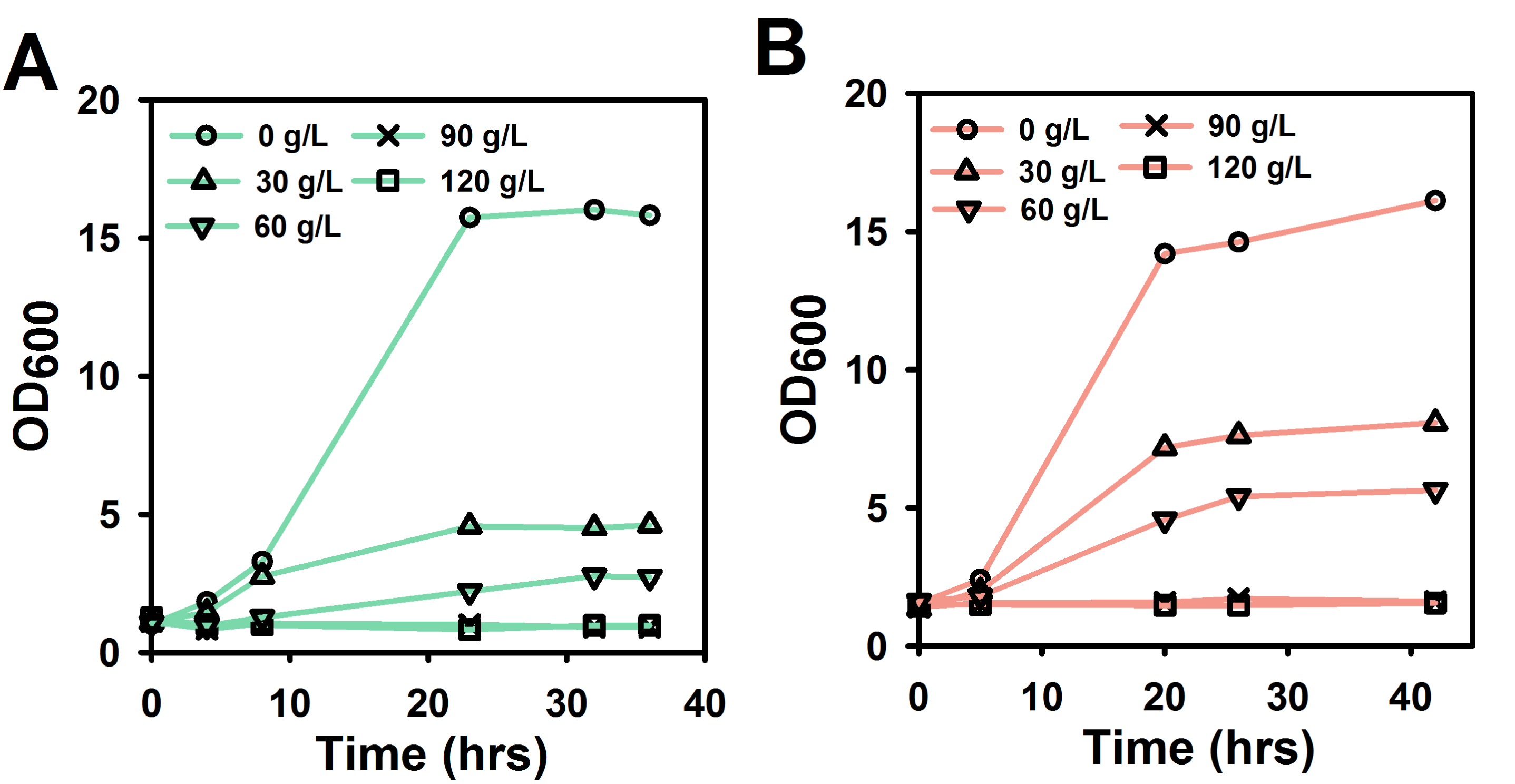

Supplement: S1 Fig — Effects of initial ethanol concentrations on cell growth of S. cerevisiae (A) EJ2 and (B) SR8. The inoculum size is 0.45 g dry cell weight/L and initial concentrations of cellobiose and xylose are 40 g/L. (TIF) [file pone.0199104.s003.tif]

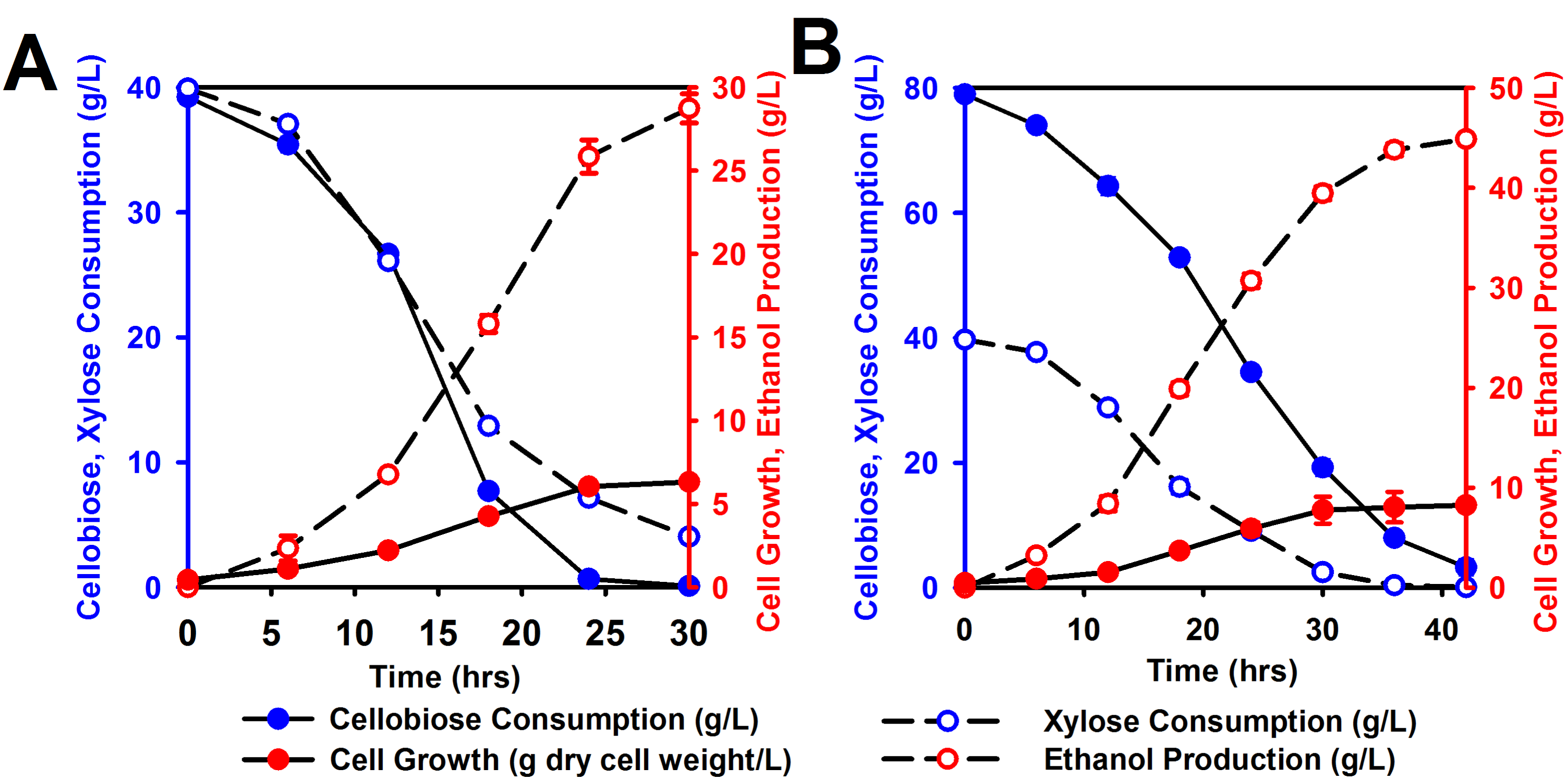

Supplement: S2 Fig — Experimental profiles of cell growth, cellobiose consumption, xylose consumption and ethanol production at different initial sugar concentrations using S. cerevisiae strain ES. The initial sugar concentrations are 40 g/L cellobiose+40 g/L xylose (A) and 80 g/L cellobiose+ 40 g/L xylose (B). The inoculum size is 0.45 g dry cell weight/L. Results are the means of duplicate experiments; error bars indicating standard deviations are not visible when smaller than the symbol size. (TIF) [file pone.0199104.s004.tif]

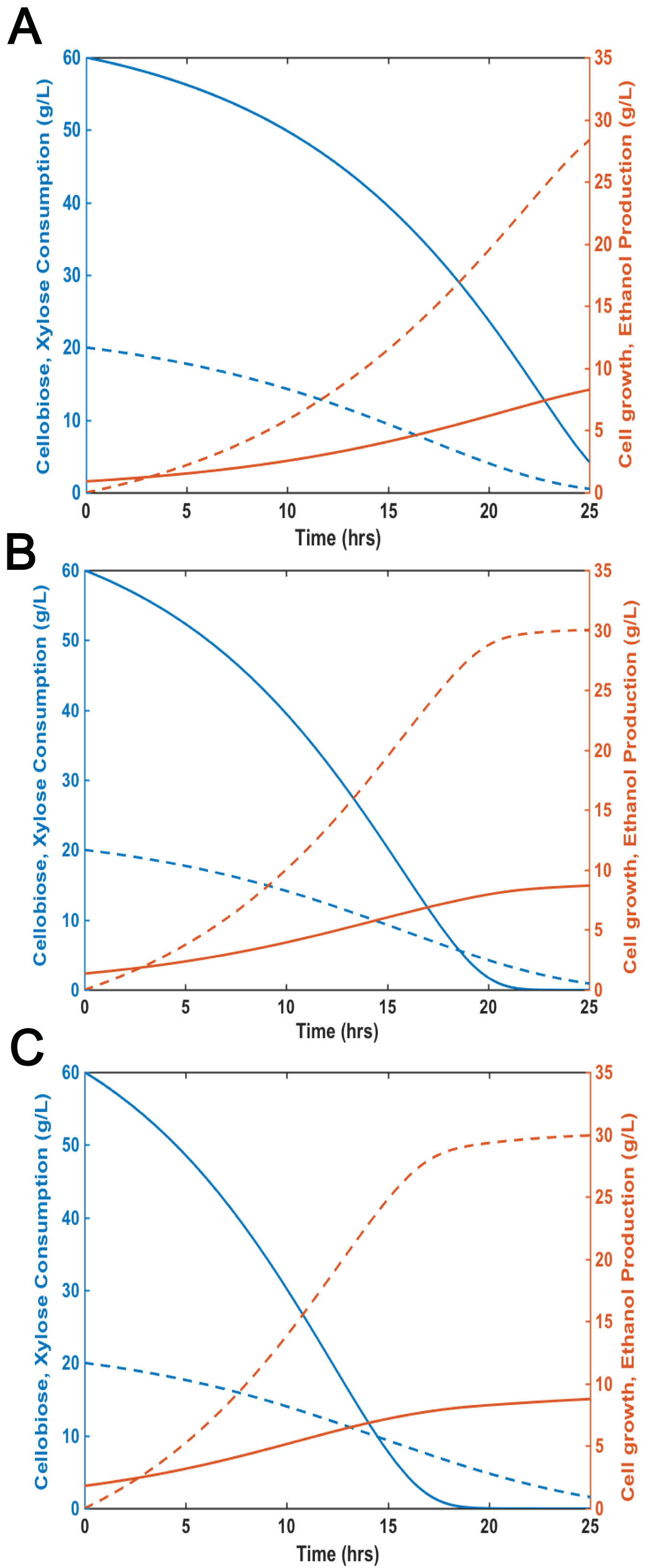

Supplement: S3 Fig — Model simulated profiles of cell growth, cellobiose consumption, xylose consumption and ethanol production at initial sugar concentrations and inoculum sizes using S. cerevisiae strain EJ2 and SR8. The initial sugar concentrations are 60 g/L cellobiose+20 g/L xylose. The initial cell densities of EJ2+SR8 were 0.45 g dry cell weight/L+0.45 g dry cell weight/L (A), 0.9 g dry cell weight /L+0.45 g dry cell weight /L (B) and 1.35 g dry cell weight /L+0.45 g dry cell weight /L (C). Lines represent model predictions (blue solid line, model curve of cellobiose consumption; blue dash line, model curve of xylose consumption; red solid line, model curve of cell growth; red dash line, model curve of ethanol production). (TIF) [file pone.0199104.s005.tif]
